# Assessment of aortic valve in regard to its anatomical variants morphology in 2053 patients using 64-slice CT retrospective coronary angiography

**DOI:** 10.1186/s12872-016-0261-z

**Published:** 2016-05-10

**Authors:** Konrad Szymczyk, Michał Polguj, Ewa Szymczyk, Leopold Bakoń, Ryszard Pacho, Ludomir Stefańczyk

**Affiliations:** Department of Radiology, Barlicki University Hospital, Medical University of Lodz, ul. Kopcińskiego 22, 90-153 Lodz, Poland; Department of Angiology, Medical University of Lodz, Narutowicza 60, Łódź, 90-136 Poland; II Department of Clinical Radiology, Medical University of Warsaw, ul. Banacha 1A, 02-097 Warsaw, Poland

**Keywords:** Bicuspid, Unicuspid, Quadricuspid aortic valve, Coronary computed tomography angiography

## Abstract

**Background:**

Bicuspid aortic valve (BAV) is the most common congenital cardiac anomaly. Other aortic valve variants are rare but are associated with an increased incidence of various pathologies of the aortic valve (AV). The aim of this study was to assess the AV function in regard to its anatomical variants morphology in patients who underwent 64-slice coronary computed tomography angiography (CCTA) for suspected or known coronary artery disease.

**Methods:**

The results of 64-detector retrospective ECG-gated CCTA of 2053 patients (mean age 58 years; 1265 males) were analyzed retrospectively by experienced cardiovascular radiologist. Coronary anatomy (with coronary artery dominance) and the extent of occlusion in the coronary arteries were assessed. Furthermore morphological and functional status of AV variants were analyzed. Among measured parameters were area at the level of AV annulus, orifice and tubular portion of the ascending aorta.

**Results:**

The AV was visualized in all CCTA studies and the analysis of its morphology and function was done in all patients. BAV was found in 19 patients (0.9 %), from which type 0 was diagnosed in five patients (0.2 %) and type 1 in 14 patients (0.7 %) - there was no patient with BAV type 2. Unicuspid (UAV) and quadricuspid (QAV) variant were both observed each in one patient (0.05 %). In rest of the patients from the study group tricuspid AV variant was recognized. Function of AV variants was mostly affected in BAV0 and UAV. Among patients with BAV1 there were patients with normal and abnormal function of AV. QAV variant did not deteriorate AV function. There was no difference in coronary artery disease and dominancy between different anatomical variants of AV.

**Conclusions:**

During CCTA different valve variants can be detected and detailed analysis of valvular function can be proceeded. Larger values of annulus area, wider diameters of ascending aorta and more stenotic profile were observed in BAV 0, BAV 1 and UAV. Among AV variants morphology and function was mostly affected in patients with BAV 0 and UAV variants, while subjects with BAV1 had normal or abnormal function of the AV. Moreover, we noticed that QAV variant did not deteriorate AV function.

**Electronic supplementary material:**

The online version of this article (doi:10.1186/s12872-016-0261-z) contains supplementary material, which is available to authorized users.

## Background

Bicuspid aortic valve (BAV) is the most common congenital cardiac anomaly, with an estimated incidence of 0.9 to 2 % in the general population [1]. BAV is a well known risk factor for aortic dilatation and acute aortic dissection, which is related to underlying aortopathy, cystic medial degeneration, and hemodynamic factors. Other aortic valve variants eg. unicuspid (unicommissural) and quadricuspid aortic valve are extremely rare and can be associated with an increased incidence of various pathologies (e.g. stenosis, regurgitation, endocarditis, aneurysmal dilatation of the ascending aorta and aortic dissection) which usually become symptomatic at an earlier age [2–4].

Currently, coronary computed tomography angiography (CCTA) in not only the alternative to invasive angiography in the evaluation of coronary anatomy, but also allows concomitant precise evaluation of other cardiac or vascular structures, especially aortic valve anatomy what could be crucial for detecting aortic valve variants [5, 6].

The aim of this study was to assess the aortic valve function in regard to its anatomical variants morphology in patients who underwent 64-slice CCTA for suspected or known coronary artery disease.

## Methods

### Patients

The results of ECG-gated CCTA in 2053 patients (mean age 58 years; 1265 males and 788 females) were analyzed retrospectively. All examinations were performed from December 2010 to June 2015. All data analysed were collected as part of routine diagnosis according to national guidelines and agreements. All procedures took place in accordance with the ethical standards of the responsible committee on human experimentation (institutional and national) and with the Helsinki Declaration of 1975, as revised in 2008. The research project was approved also by the Bioethics Commission of the Medical University of Lodz (protocol No. RNN/28/16/KE). The main indications for CCTA were: detection of coronary artery disease in symptomatic patients without known heart disease; preoperative coronary assessment prior to noncoronary cardiac surgery; patients with prior electrocardiographic exercise testing - normal test with continued symptoms or intermediate risk Duke treadmill score; patients with prior stress imaging procedures - discordant electrocardiographic exercise and imaging results or equivocal stress imaging results; assessment post-revascularization in patients with stents >3 mm and clinical presentation suggesting low-to-intermediate probability for restenosis; evaluation of bypass graft patency. Exclusion criteria for CCTA were arrhythmia (e.g., atrial fibrillation or flutter, frequent irregular premature ventricular contractions or premature atrial contractions); previous serious allergic reaction to iodine contrast medium, renal failure (GFR < 60 ml/min/1,73 m2); pregnancy; obesity with body mass index > 40 kg/m2; heart rate > 70 beats per minute refractory to heart-rate lowering agents, high coronary calcium score result (>800 A.U.). According to ESC guidelines in patients with calcium score over 400 A.U. calcified plaque distribution was analyzed. If plaques were diffused and small enabling the reliable arterial lumen analysis, CCTA was performed despite calcium score over 400 A.U. Due to authors previous experience calcium score over 800 A.U. do not prognose favorable plaque distribution and was consider as contraindication for CCTA, unless it was patient with coronary artery bypass graft and known advanced coronary artery disease and in whom evaluation of graft patency was main target.”

### Computed tomography protocols and image reconstruction

All studies were performed using a 64-detector CT scanner with retrospective ECG gating (Lightspeed VCT, GE Healthcare, Milwaukee, WI, US). In all patients with suspected coronary atherosclerotic disease coronary artery calcium score (CACS) was determined. Coronary CT angiography was not performed if CACS was over 800 A.U, unless patient was referred for determination of bypass grafts patency. In the rest of patients, complete CT evaluation was performed with the administration of a contrast agent. If there was no contraindication, intravenous beta-blocker (up to total dose of 20 mg metoprolol) was administered just before the scan if the heart rate was greater than 70 beats per minute. The heart rate of patients ranged between 50 and 97 beats per minute (mean 66 beats per minute). An intravenous cannula was placed in the right basilica vein. The triphasic injection protocol was used with the injection of 60–70 mL of non-ionic contrast medium with high iodine concentration (iomeprol 400 mgI/mL, iomeron) at a flow rate of 4.8–5.2 mL/s followed by injection of a 40 mL solution of 20 % contrast medium and 80 % saline solution, and finally an injection of 30 mL saline with the same flow rate was used for CCTA.

### Imaging results analysis

**Fig. 1:**
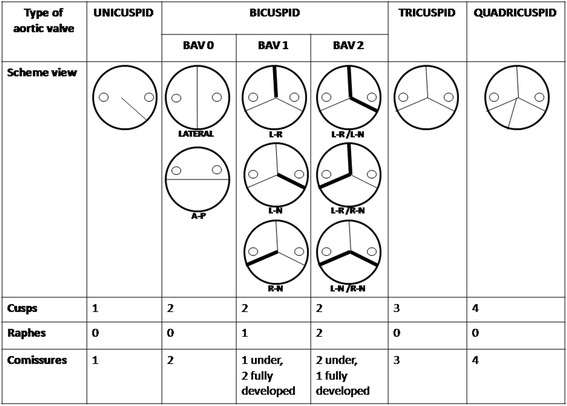
Review of possible anatomic variants of aortic valves. BAV – bicuspid aortic valve, A-P – anteroposterior, L-R – left-right, L-N – left-noncoronary, R-N – right-noncoronary. Adapted from Angelini et al. [7] and Sievers et al. [8]

CT scans were processed and analyzed off-line on a dedicated workstation (Advantage Workstation 4.4, GE Healthcare, Milwaukee, WI, US) using curved multiplanar reconstruction and 3D volume rendering reconstructions. To evaluate the aortic valve morphology, multiphase reconstructions at 10 % intervals of R-R were created from 0 to 90 % (usually 20 % for the midsystolic window and 70 % for the diastolic phase in passive ventricular filling). The morphology of the valve leaflets was assessed in the reconstructed oblique plane parallel to the aortic annulus in the systolic and diastolic phases. All examinations were reviewed by experienced radiologist. Coronary anatomy (with coronary artery dominance) and the extent of occlusion in the coronary arteries were assessed. Furthermore, morphological and functional status of aortic valve were assessed. Similarly to Angelini et al. [7] and Sievers et al. [8] in our research the classification system of aortic valve anatomy was based on three major features: (1) the number of raphes, (2) the spatial position of cusps or raphes, and (3) the functional status of the valve. Aortic valves were classified as unicuspid (UAV), bicuspid (BAV), tricuspid (TAV) or quadricuspid (QAV). Moreover according to the number of raphes the bicuspid aortic valves were described as *type 0* - valve with no raphe (the orientation of the free edge of the cusps was found to be either anteroposterior or lateral), *type 1* - valves with one raphe and *type 2* - valves with two raphes (in both types the orientation of the raphes in relation to the sinuses defined the subcategory). Schematic variants of aortic valve are presented in Fig. 1.

Among other analyzed parameters were aortic maximal diameters and areas at the level of annulus, orifice and tubular portion of the ascending aorta. All parameters were measured in the diastolic phase of cardiac cycle. Moreover, we compared values of two indexes reflecting the degree of valvular stenotic profile. First index reflected ratio [(Annulus - Orifice)/Annulus] and second index reflected ratio [Orifice/Annulus] in different aortic anatomical variants.

Statistical analysis of data was performed using MedCalc version 9.5.2.0 (MedCalc Software, Frank Schoonjans 1993–2008, Belgium). Continuous variables are presented as mean ± SD and compared using the 2-tailed, unpaired Student t test. The 2-tailed probability value of *P* < 0.05 was considered statistically significant.

The research protocol was approved by the local bioethics committee. All data analyzed were collected as part of routine diagnosis according to national guidelines and agreements.

If ethics was not required for your study, then this should be clearly stated and a rationale provided.

## Results

**Fig. 2:**
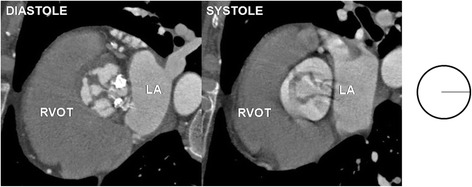
Unicuspid aortic valve - single lunar shape leaflet and single commissure. Note small orifice area responsible for stenotic dysfunction of the valve. RVOT – right ventricular outflow tract, LA – left atrium

**Fig. 3:**
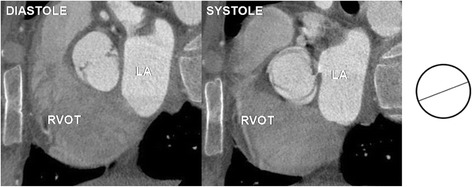
Bicuspid aortic valve type 0 – two leaflets, two commissures, no raphe. RVOT – right ventricular outflow tract, LA – left atrium

**Fig. 4:**
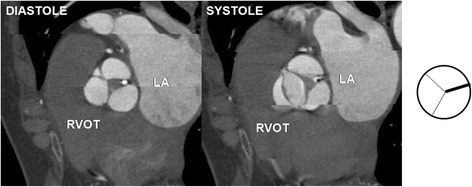
Bicuspid aortic valve type 1- two leaflets, two fully developed commissures, single underdeveloped commissure with raphe. Note small orifice area responsible for stenotic dysfunction of the valve. RVOT – right ventricular outflow tract, LA – left atrium

**Fig. 5:**
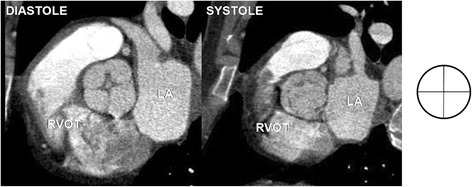
Quadricuspid aortic valve - four leaflets, four commissures. RVOT – right ventricular outflow tract, LA – left atrium

The aortic valve was clearly visualized in all coronary computed tomography angiography studies and the analysis of its morphology and function was possible in all patients from the study group. Bicuspid aortic valves were found in 19 patients (0,9 %), from which type 0 was diagnosed in five patients (0,2 %) and type 1 in 14 patients (0,7 %) - there was no patient with BAV type 2. Unicuspid and quadricuspid variant were both observed only in one patient (0,05 %). In rest of the patients from the study group tricuspid aortic valve variant was recognized. For the comparison of morphology and function of tricuspid aortic valve consecutive first 24 patients were selected. Examples of unicuspid, bicuspid, tricuspid and quadricuspid valves are presented in Figs. 2, 3, 4 and 5.

Baseline characteristic of different types of aortic valve variants are presented in Table 1.

**Table 1:** Baseline characteristic of aortic valve variants

ANNULUS					
Parameter	UAV (*n* = 1)	BAV 0 (*n* = 5)	BAV 1 (*n* = 14)	TAV (*n* = 24)	QAV (*n* = 1)
Mean [mm^2^]	835,00	730,40	696,00	546,71	756,00
SD [mm^2^]	-	198,00	227,12	96,23	-
Min [mm^2^]	835,00	380,00	404,00	413,00	756,00
Max [mm^2^]	835,00	848,00	1223,00	713,00	756,00
ORIFICE					
Parameter	UAV (*n* = 1)	BAV 0 (*n* = 5)	BAV 1 (*n* = 14)	TAV (*n* = 24)	QAV (*n* = 1)
Mean [mm^2^]	262,00	401,20	329,43	357,96	562,00
SD [mm^2^]	-	293,96	167,53	76,85	-
Min [mm^2^]	262,00	165,00	176,00	239,00	562,00
Max [mm^2^]	262,00	765,00	774,00	493,00	562,00
INDEX = (Annulus - Orifice)/Annulus					
Parameter	UAV (*n* = 1)	BAV 0 (*n* = 5)	BAV 1 (*n* = 14)	TAV (*n* = 24)	QAV (*n* = 1)
Mean [−]	0,69	0,46	0,52	0,34	0,26
SD [−]	-	0,32	0,18	0,11	-
Min [−]	0,69	0,09	0,02	0,06	0,26
Max [−]	0,69	0,79	0,73	0,55	0,26
ORIFICE/ANNULUS					
Parameter	UAV (*n* = 1)	BAV 0 (*n* = 5)	BAV 1 (*n* = 14)	TAV (*n* = 24)	QAV (*n* = 1)
Mean [−]	0,31	0,54	0,48	0,66	0,74
SD [−]	-	0,32	0,18	0,11	-
Min [−]	0,31	0,21	0,27	0,45	0,74
Max [−]	0,31	0,91	0,98	0,94	0,74
ASCENDENS					
Parameter	UAV (*n* = 1)	BAV 0 (*n* = 5)	BAV 1 (*n* = 14)	TAV (*n* = 24)	QAV (*n* = 1)
Mean [mm]	54,00	35,20	43,29	30,88	39,00
SD [mm]	-	9,36	10,06	2,76	-
Min [mm]	54,00	24,00	27,00	27,00	39,00
Max [mm]	54,00	46,00	70,00	38,00	39,00

BAV – biscupid aortic valve, UAV – unicuspid aortic valve, QAV – quadricuspid aortic valve, TAV – tricuspid aortic valve, Index - (annulus - orifice)/annulus

Among patients with BAV aortic valve regurgitation was recognized in 4 pts (two with BAV 0 and two with BAV 1). Stenotic profile of aortic valve was found in two patients with BAV 1 variant. There was no difference in coronary artery disease and dominancy between different anatomical variants of the aortic valve.

**Fig. 6:**
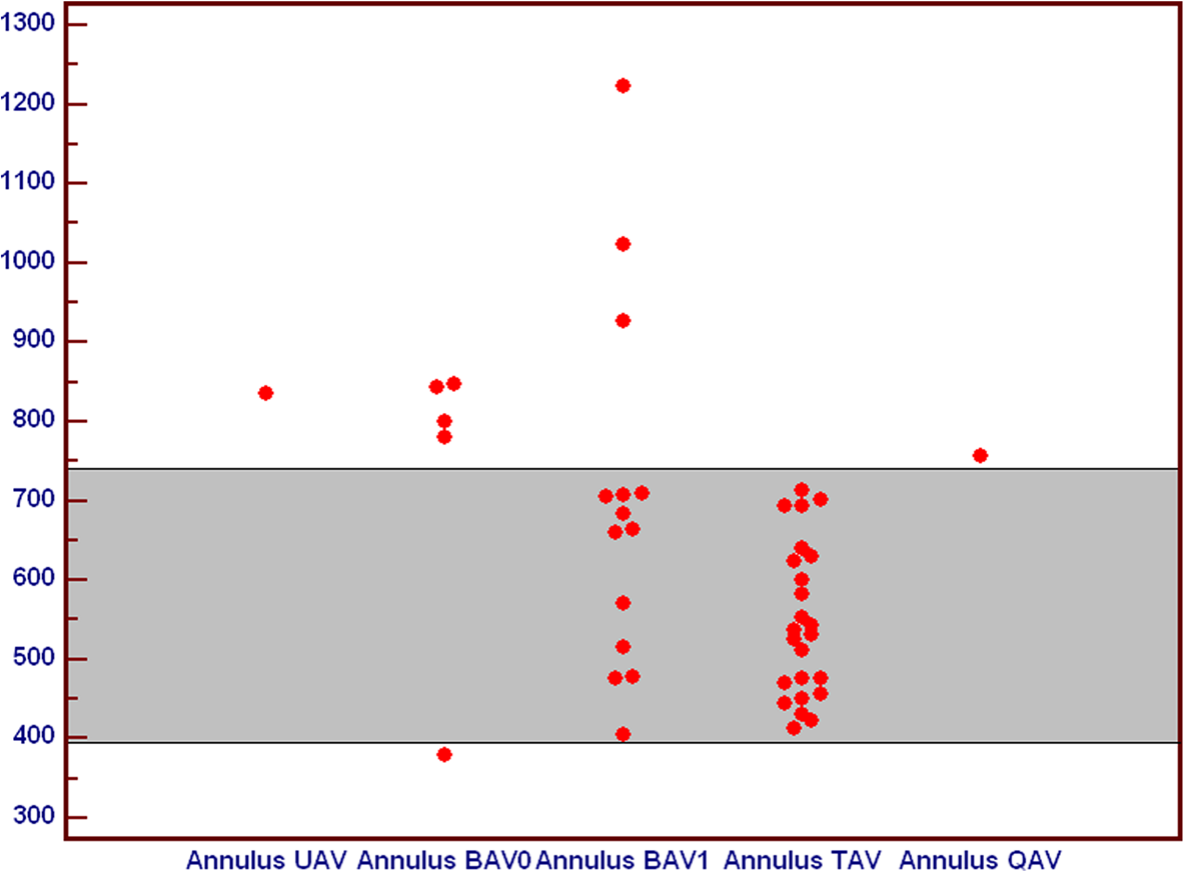
Comparison of annulus area in subgroups of patients with unicuspid (UAV), bicuspid (BAV), tricuspid (TAV) and quadricuspid (QAV) aortic valve. All values are in [mm^2^]. BAV – bicuspid aortic valve, QAV – quadricuspid aortic valve, TAV – tricuspid aortic valve, UAV – unicuspid aortic valve

Comparing areas of AV annulus among different aortic valve anatomical variants, we observed that annulus areas of BAV 0 (*P* = 0,004), BAV 1 (*P* = 0,008) and UAV were significantly larger than in TAV, while annulus area of TAV and QAV annulus were similar. In patients with BAV1 variant there was a wide spectrum of annulus areas with values comparable to those observed in TAV but also much larger values (Fig. 6).

**Fig. 7:**
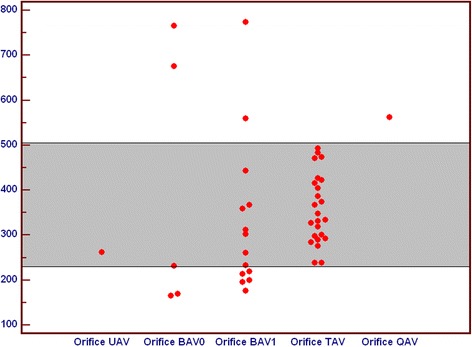
Comparison of orifice area in subgroups of patients with unicuspid (UAV), bicuspid (BAV), tricuspid (TAV) and quadricuspid (QAV) aortic valve. All values are in [mm^2^]. BAV – bicuspid aortic valve, QAV – quadricuspid aortic valve, TAV – tricuspid aortic valve, UAV – unicuspid aortic valve

Comparing orifice areas of different aortic valve variants, we noticed that among BAV 0 there were no values similar to TAV, only small and large values of orifice areas were observed. Orifice areas for TAV and QAV were comparable, while orifice in UAV was stenotic. Among BAV1 there was a wide spectrum of orifice areas, ranging from stenotic through normal to large profile (Fig. 7).

**Fig. 8:**
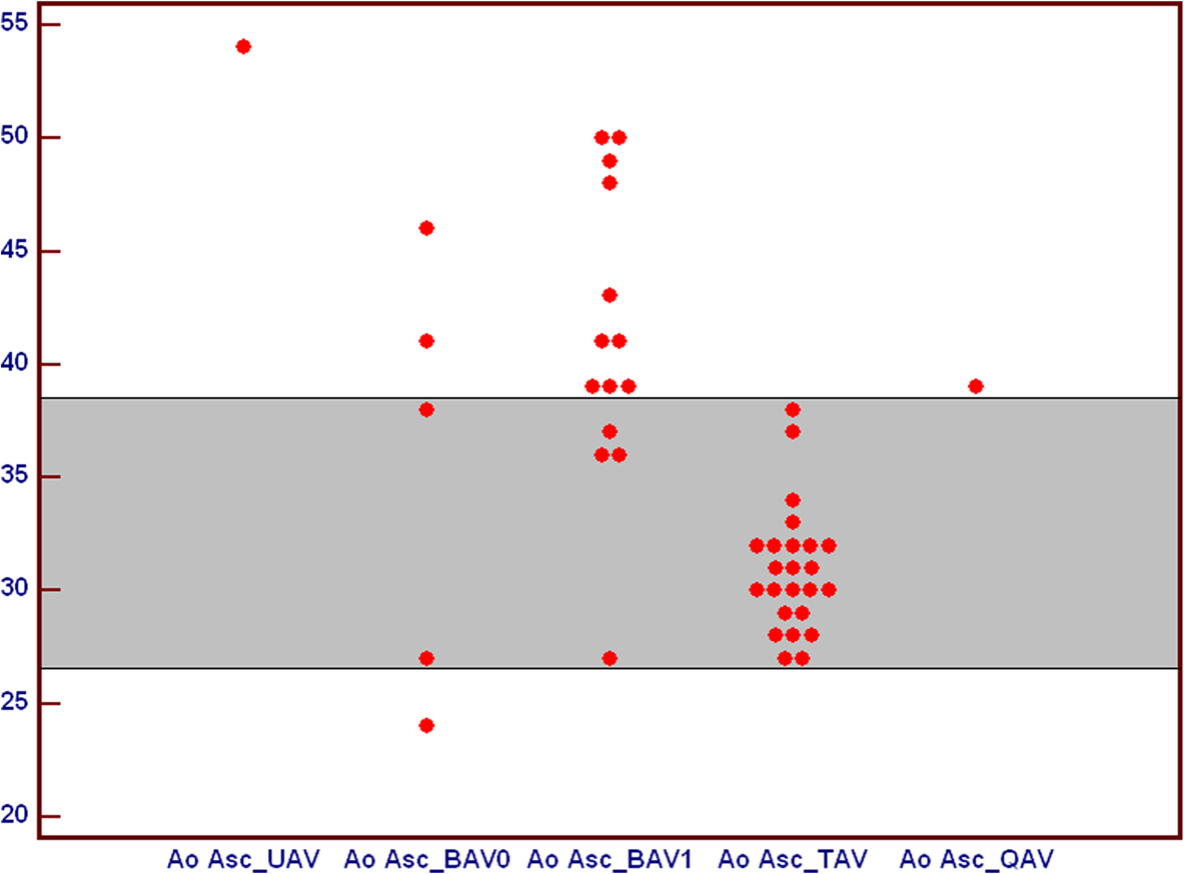
Comparison of ascending aorta diameter in subgroups of patients with unicuspid (UAV), bicuspid (BAV), tricuspid (TAV) and quadricuspid (QAV) aortic valve. All values are in [mm]. BAV – bicuspid aortic valve, QAV – quadricuspid aortic valve, TAV – tricuspid aortic valve, UAV – unicuspid aortic valve

Moreover we noticed wider diameter of ascending aorta in UAV. Ascending aorta diameter for TAV and QAV were comparable. Among BAV1 and BAV 0 there was also wide range of ascending aorta areas (Fig. 8).

**Fig. 9:**
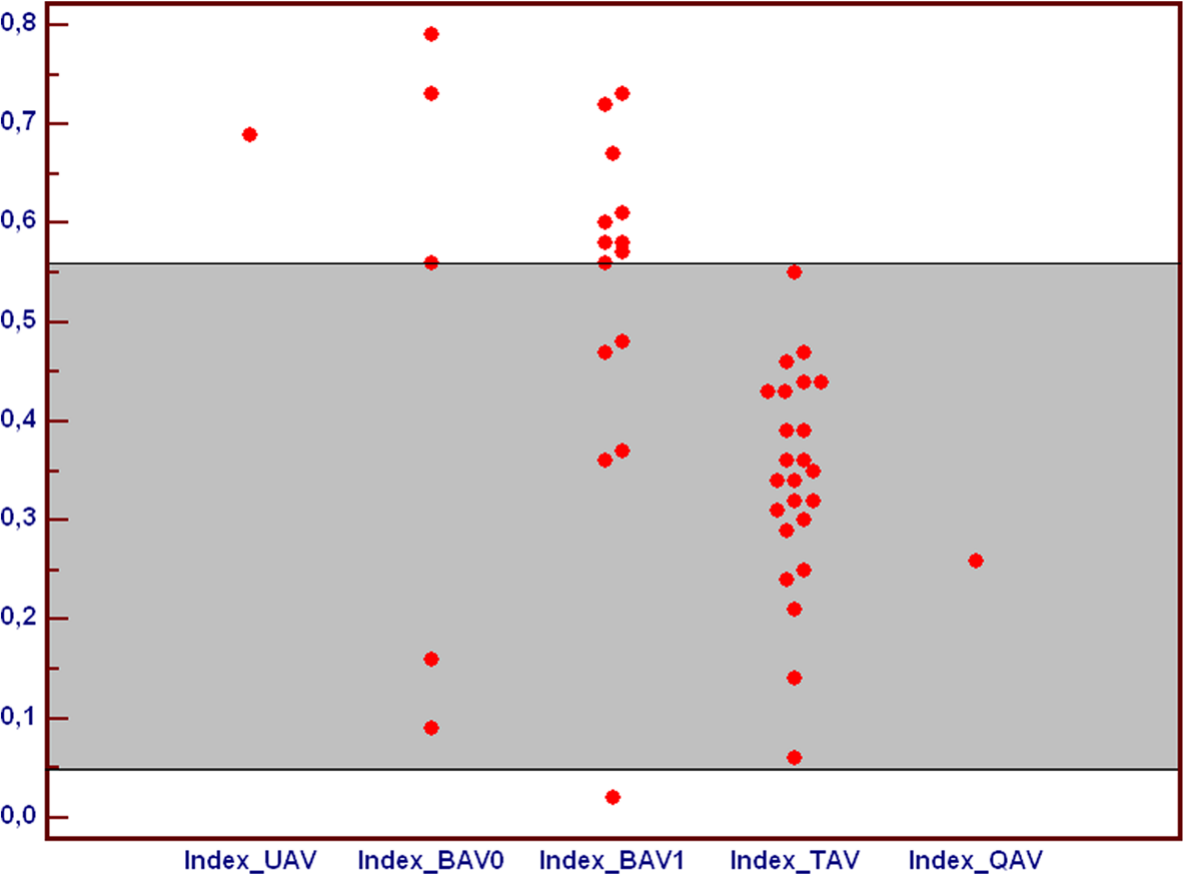
Comparison of index ([Annulus – Orifice]/Annulus) in subgroups of patients with unicuspid (UAV), bicuspid (BAV), tricuspid (TAV) and quadricuspid (QAV) aortic valve. All index values are abstract numbers. BAV – bicuspid aortic valve, QAV – quadricuspid aortic valve, TAV – tricuspid aortic valve, UAV – unicuspid aortic valve

Higher values of (Annulus-Orifice)/Annulus index related to more stenotic valve profile were observed in patients with unicuspid and both types of bicuspid aortic valve, what is related to more stenotic valve profile, while value for quadricuspid valve was comparable to tricuspid valves (Fig. 9).

**Fig. 10:**
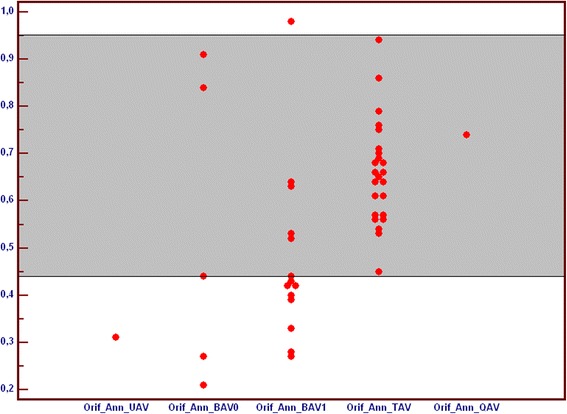
Comparison of index [Orifice/Annulus] in subgroups of patients with unicuspid (UAV), bicuspid (BAV), tricuspid (TAV) and quadricuspid (QAV) aortic valve. All index values are abstract numbers. BAV – bicuspid aortic valve, QAV – quadricuspid aortic valve, TAV – tricuspid aortic valve, UAV – unicuspid aortic valve

Smaller values of Orifice/Annulus index related to more stenotic valve profile were observed in patients with unicuspid and both types of bicuspid aortic valve, while value for quadricuspid valve was comparable to tricuspid valves (Fig. 10).

Taking into account all analyzed parameters we observed that function of aortic valve variants was mostly affected in BAV0 and UAV. Among patients with BAV1 there were patients with normal and abnormal function of the aortic valve. In our study we noticed that QAV variant did not deteriorate aortic valve function.

## Discussion

While MDCT has been widely established for assessment of coronary arteries, the cardiac valves are routinely assessed by transthoracic and transesophageal echocardiography. Due to limitations of echocardiography, such intra- and interobserver variability, its flow dependency and high patient-related differences in image quality, the multimodality imaging approach, including MDCT, could be crucial for diagnostic workup of valvular disease. This study showed that aortic valve morphology and function as well as proximal part of ascending aorta can be routinely assessed by ECG gated 64 slice computed tomography during non-invasive coronary artery evaluation. Further progress in temporal and spatial resolution with concomitant reduction of radiation dose may overcome some current limitations and broaden role of CCTA in aortic valve assessments, especially before planned interventions (e.g. TAVI).

Among congenital cardiac anomalies the bicuspid aortic valve is the most common pathology, with an estimated incidence of 0.9 to 2 % in the general population [1]. Other aortic valve variants are extremely rare with the incidence of 0.02 % for unicuspid (unicommissural) and 0.013 % for quadricuspid aortic valve [2, 3]. Other than tricuspid morphology of aortic valve is associated with an increased incidence of various pathologies (e.g. stenosis, regurgitation, endocarditis, aneurysmal dilatation of the ascending aorta and aortic dissection) which usually become symptomatic at an earlier age [3, 4]. In this study BAV was found in 0.9 % (0.2 % BAV 0 and 0.7 % BAV 1), while both UAV and QAV were observed each in one patient (0.05 %).

There was no difference in coronary artery disease and dominancy between different anatomical variants of aortic valve.

We observed that function of aortic valve variants was mostly affected in BAV0 and UAV. Among patients with BAV1 there were patients with normal and abnormal function of the aortic valve. In our study we noticed that QAV variant did not deteriorate aortic valve function.

In BAV 1 and BAV 0 we observed smaller orifice and annulus areas with wider aortic diameter, what is concordant with previous studies and is important for clinical practice, because aortic dilatation has a propensity for dissection and rupture, making it a potentially lethal disease [9, 10]. Ascending aortic dilatation with BAV warrants frequent monitoring, with possible early prophylactic surgical intervention to prevent dissection or rupture. In our study we also observed that larger orifices and areas of TAV were associated with wider aortic diameter, what can be explained with proper relation between aortic valve and ascending aorta with increasing body surface area.

Indexes incorporated to our study ”(Annulus-Orifice)/Annulus” and “Orifice/Annulus” can be used in clinical settings for assessing valve profile in different anatomical variants, especially in patients with problematic evaluation of hemodynamic importance or aortic valve stenosis in echocardiography or cardiac magnetic resonance.

Precise assessment of morphology and function of aortic valve is crucial in process of qualifying patients for aortic valve replacement surgery and less invasive percutaneus interventions. According to the guidelines of European Society of Cardiology on the management of valvular heart disease from year 2012 MDCT may contribute to the evaluation of the severity of the aortic valve disease, either indirectly by quantifying valvular calcification, or directly through the measurement of valve planimetry. Moreover it is widely used to assess the severity and location of an aneurysm of the ascending aorta. In the process of evaluating before cardiac surgery, due to its high negative predictive value, MDCT may be useful in excluding CAD in patients who are at low risk of atherosclerosis. MDCT plays an important role in the work-up of high-risk patients with aortic stenosis considered for transcatheter aortic valve implantation (TAVI) [11].

## Study limitations

Several limitations of this study must be considered. First, due to a small number of cases (especially with UAV and QAV, which are rare findings), further investigations are required to confirm our conclusions. Second, we performed only anatomical retrospective analysis of different variants of aortic valve without functional assessment of parameters derived from echocardiography or CMR.

## Conclusions

During CCTA different valve variants can be detected and detailed analysis of valvular function can be proceeded what is crucial for establishing management of valvular heart disease. Larger values of annulus area, wider diameters of ascending aorta and more stenotic profile were observed in BAV 0, BAV 1 and UAV. Among aortic valve variants morphology and function was mostly affected in patients with BAV 0 and UAV variants, while subjects with BAV1 had normal or abnormal function of the aortic valve. Moreover, we noticed that QAV variant did not deteriorate aortic valve function. However due to a small number of cases (especially with UAV and QAV), further investigations are required to confirm these findings.

## Availability of data and materials

The datasets supporting the conclusions of this article are included within the article (and its Additional file 1).

## Additional file

Additional file 1: Matedial data. (XLS 22 kb)
